# Gleanings

**Published:** 1896-09

**Authors:** 


### GLEANINGS.

An outbreak of an anasarcous character, involving the thoracic cavity very largely, is reported among the sheep of a district in Australia.

The *Live Stock Journal* reports four deaths among horses from swallowing excessive quantities of sand with drinking-water, especially when allowed to drink from shallow ponds. In one instance, on autopsy, fourteen pounds of sand were found in the digestive tract.

The control of fleas and other parasites has given little trouble at the Philadelphia Veterinary Sanitarium during the warm weather. The cats and dogs have been given creolin baths occasionally, and crude sanitas disinfecting fluid has been used on the walls and floors, and the kennels have been thoroughly washed with hot water freely charged with sanitas.

The *Holstein Friesian Register* quotes Professor King, of the Wisconsin Experiment Station, as authority for the statement that fourteen hundred cubic feet of air per hour should move into and out of the stables for each cow. Outlet shafts of one foot square, inside measure, are requisite for every ten cows. Outlet shafts should start near the floor and be carried well above the roof of the barn.

Calcium chloride will be well worth a trial in those cases of skin diseases of dogs where the itching and irritation of the skin is so intense and pronounced. Give in water in from one- to ten-grain doses after feeding until the blood is well charged with the remedy.

The fluidextract of gelsemium, with Norwood's tincture of veratrum viride, secured a favorable result after sixteen days' treatment in a case of tetanus in a boy suffering from a wound of the foot from broken glass. The case is reported in the *Medical News* of July 18, 1896.

A preparation of formalin-gelatin as an antiseptic powder for wounds and in operative surgery has recently been introduced under the name of glutol. It is odorless, and is claimed to be absolutely non-irritant and non-poisonous. In open wounds it limits the formation of pus and aids the elimination of dead tissue. In moist wounds its absorbent qualities are said to be very marked, and in experimental abdominal surgery it has demonstated some remarkable properties which promise to make it an invaluable preparation in this sphere of work. Veterinarian George Rodewald, of Germany, quotes a rapid healing of a suppurative abdominal wound in a fox-terrier. The wound was cleansed daily with a lysol solution and dusted with glutol. It is also claimed by Veterinarian Jess, of Charlottenburg, to allay the itching in wounds of dogs which so often proves a great barrier to healing. The same writer reports a case of broken knees in a colt rapidly healing after cleansing with creolin solution and dusting with glutol, without the application of bandages.

William Watson, who has been writing a series of articles on abortion, has finally arrived at the conclusion that it may be infectious, but disclaims its contagious character. He is now convinced that matter discharged from the genitals may convey the disease to other animals, or it may be conveyed by the hands or instruments of attendants.

One of the most common ailments of pet house-cats is the formation of hair-balls along the intestinal tract, causing the most obstinate torpidity of the bowels. It is largely the result of overfeeding, which produces a dry condition of the hair, which enters the mouth and stomach when the animal washes itself. Its dry condition facilitates its impeding the natural movements of the bowels. A cat recently treated in the Philadelphia Veterinary Sanitarium, in which the obstruction could be felt by external manipulation, continued to refuse food for ten days. Repeated doses of castor-oil and the introduction of gluten suppositories finally brought away five large masses which were made up almost entirely of matted hair.

The *Breeders' Gazette*, in a recent number, invites its readers and others to a discussion of the legal points involved in the sale of an animal guaranteed to be a breeder, and which has proved to be only a potent sire or dam in occasional instances.

The average yearly consumption of whole milk per inhabitant of the United States is said to be twenty-five and one-half gallons, and of butter nearly twenty pounds. Does anyone presume to say that these consumers have no right to ask that these products shall come from healthy animals and that these foods shall be gathered and disposed of to the consumers under the most rigid care as to cleanliness and purity?

